# Neutrophil extracellular traps: the hidden driver of gastrointestinal cancer and emerging therapeutic strategies

**DOI:** 10.3389/fimmu.2026.1793013

**Published:** 2026-04-16

**Authors:** Yujing Zhou, Hui Shan, Yifei Pei, Wen Zhang, Min Xu

**Affiliations:** 1 Department of Gastroenterology, Affiliated Hospital of Jiangsu University, Jiangsu University, Zhenjiang, China; 2 Department of Oncology, Zhenjiang First People’s Hospital, Zhenjiang, China; 3 Department of Microbiology, School of Medicine, Jiangsu University, Zhenjiang, China

**Keywords:** cancer immunity, gastrointestinal tumors, neutrophil extracellular traps, targeted therapy, tumor microenvironment

## Abstract

As the most abundant subset of leukocytes in the innate immune system, neutrophils are central to host defense, particularly against bacterial and fungal infections. With advances in tumor microenvironment (TME) research, their multifunctional roles in tumor biology have been clarified, and the identification of neutrophil extracellular traps (NETs) has provided a novel perspective for understanding neutrophil-mediated regulation of tumor initiation, progression, and metastasis. NETs are reticular structures of chromatin fibers released by activated neutrophils, comprising DNA backbones, histones, and various antimicrobial proteins, initially recognized as an antibacterial defense mechanism. However, recent studies reveal NETs exert a dual role in tumor progression: directly promoting metastasis by enhancing tumor cell migration, trapping circulating tumor cells (CTCs), reactivating dormant cancer cells, and increasing vascular permeability, while also reshaping the TME to support pre-metastatic niche formation. This review systematically summarizes the molecular mechanisms of NETs in gastrointestinal tumor initiation, progression, and metastasis, and explores potential NETs-targeted anti-tumor therapeutic strategies, aiming to provide a theoretical basis for novel gastrointestinal tumor treatment directions.

## 1 Background

Neutrophils, the most abundant leukocytes in the innate immune system, are crucial first responders in host defense against bacterial and fungal infections (1–3). Recent investigations into the TME have uncovered their complex dual roles in tumor biology, with the identification of NETs representing a pivotal breakthrough that advanced understanding of neutrophil-mediated regulation of tumor progression.

NETs are web-like chromatin fiber structures released by activated neutrophils, consisting of a DNA backbone decorated with histones and various antimicrobial proteins (4, 5). Initially characterized as an antimicrobial strategy that physically entraps pathogens, NETs exert paradoxical tumor effects: they directly facilitate metastasis via enhancing tumor cell migration, capturing CTCs, reactivating dormant cancer cells, and increasing vascular permeability to promote tumor cell extravasation (6), while also remodeling the TME to create a favorable environment for pre-metastatic niche establishment (7–9).

**Figure 1:**
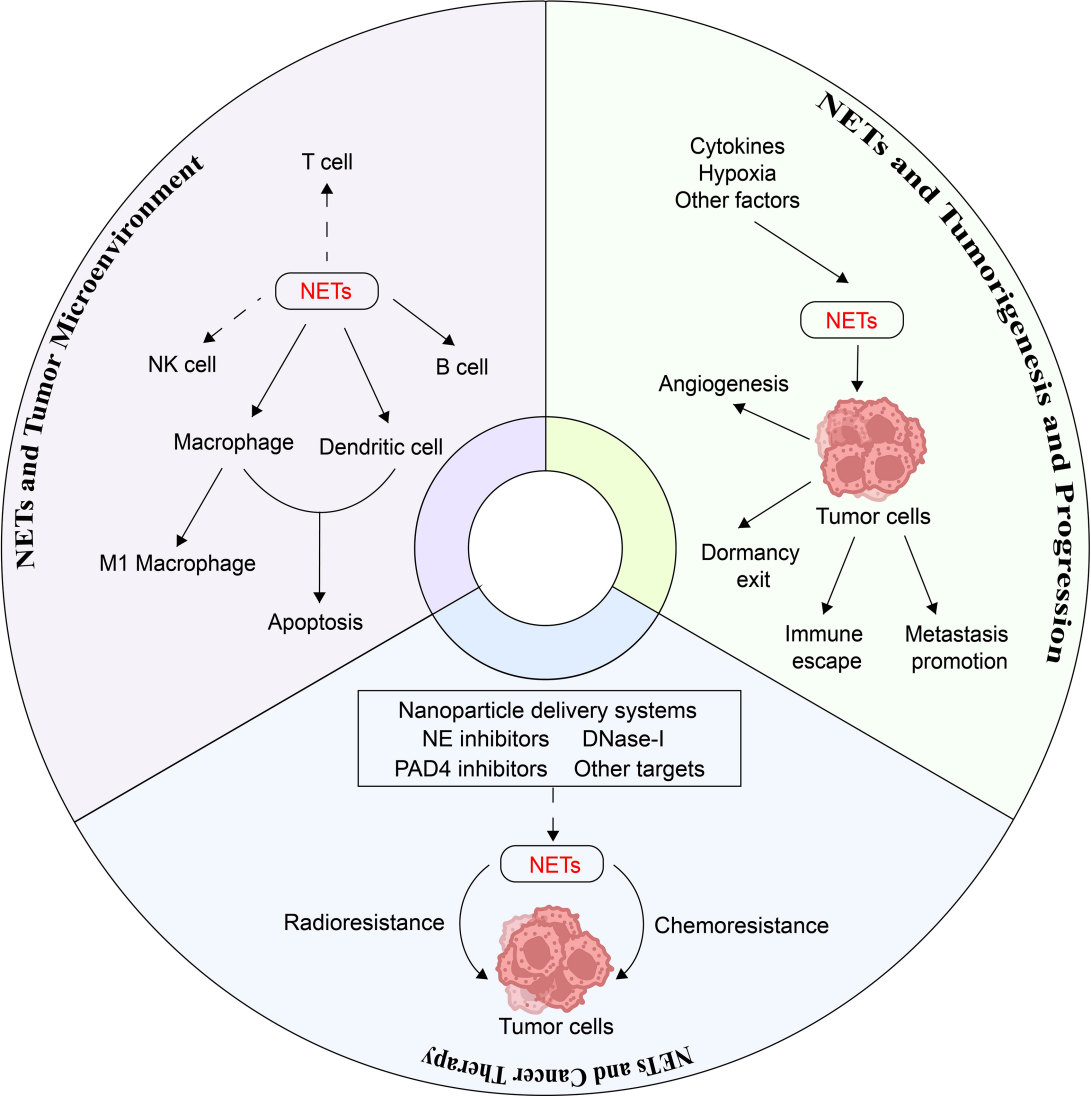
The multifaceted roles of NETs in gastrointestinal cancer. This schematic summarizes the dual role of neutrophil extracellular traps (NETs) in gastrointestinal cancer. NETs drive tumorigenesis, progression, and metastasis through multiple molecular mechanisms. Additionally, NETs contribute to therapeutic resistance by interfering with immune checkpoint inhibitors (ICIs) and promoting an immunosuppressive tumor microenvironment. Targeting NETs represents a promising therapeutic strategy for gastrointestinal malignancies.

This review delineates the molecular mechanisms of NETs in gastrointestinal tumorigenesis, progression, and metastasis, and explores potential NETs-targeted therapies to provide a theoretical framework for innovative gastrointestinal cancer treatments (**Figure 1**).

## 2 Neutrophils

Neutrophils, alternatively termed polymorphonuclear leukocytes, constitute the most abundant subset of leukocytes in the peripheral circulation. As pivotal effector cells of the innate immune system, they occupy a central position in immune defense against infections (1–3), and serve as critical components of tumor-infiltrating immune cells (10). A growing body of evidence suggests that in malignant tumors such as breast cancer and colorectal cancer, tumor-associated neutrophils (TANs) exhibit marked immunosuppressive properties and are implicated in the regulation of tumor progression (11, 12).

The Fridlender group proposed a classification framework for two functionally distinct TANs subtypes: Type N1 (anti-tumor) and Type N2 (pro-tumor) (13). Type N1 exerts anti-tumor effects primarily through the production of reactive oxygen species (ROS) and tumor necrosis factor-α (TNF-α) (14), whereas Type N2 facilitates tumor immune escape by suppressing T cell activation (15). Notably, under the influence of TME, TANs tend to differentiate toward the pro-tumor N2 phenotype (16) and can collaboratively shape an immunosuppressive microenvironment through crosstalk with various immune cell populations (17–19).

In terms of cell death modalities, neutrophils exhibit unique plasticity and can select death pathways in a microenvironment-dependent manner, including necroptosis, pyroptosis, ferroptosis, and NETosis (20). Among these, NETosis is a distinct form of programmed cell death first characterized by Brinkmann et al.: upon stimulation by pathogens, neutrophils release NETs composed of DNA fibers, granular proteins, bactericidal molecules, proteases, and histones, and this specific death process is defined as “NETosis” (21). Beyond their well-established antibacterial function, subsequent studies have demonstrated that NETs play an important regulatory role in a range of pathophysiological processes.

## 3 Formation and functions of neutrophil extracellular traps

Upon stimulation by pathogens, activated neutrophils release chromatin and granular proteins to form NETs (4). Structurally, NETs are reticular assemblies composed of decondensed chromatin fibers, histones, and granular proteins—including matrix metalloproteinase 9 (MMP9), neutrophil elastase (NE), myeloperoxidase (MPO), and cathepsin G (CG)—as well as other antibacterial proteins (5).The formation of NETs primarily involves two pathways: the NADPH oxidase (NOX)-dependent pathway and the NOX-independent pathway (22), among which ROS serves as a core regulatory molecule (23). Previous studies have shown that ROS inhibits the activity of prolyl hydroxylase (PHD), thereby stabilizing the hypoxia-inducible factor-1α (HIF-1α) protein to promote NET formation; this process also leads to the abnormal accumulation of HIF-1α in cancer cells (24, 25).

Mass spectrometry-based analyses have revealed that NETs derived from human neutrophils contain more than 500 functional proteins (26). As a key neutrophil-derived proteolytic enzyme, MMP9 can be released into the extracellular space through degranulation and binding to NETs; it participates in TME remodeling by altering the extracellular matrix (ECM) and reactivates dormant cancer cells (7). As a serine protease, NE can specifically cleave histone 4 following nuclear translocation to promote chromatin decondensation, a step widely recognized as crucial for NETs formation. Additionally, NE can regulate NETs production by degrading actin-associated cytokines (27–29). CG is a characteristic protease of azurophilic granules; in addition to its bactericidal activity, it may promote cancer cell invasion by activating metalloproteinases and hydrolyzing ECM components (30, 31). Peptidylarginine deiminase 4 (PAD4) is a neutrophil-enriched nuclear enzyme that acts on histones in a calcium-dependent manner (32). Histone modifiers also play important roles in NETosis: for example, deacetylation of histones by deacetylases is required to initiate PAD4-mediated NETs formation. Recently, cyclin-dependent kinase 4/6 (CDK4/6) has been identified as a novel regulatory node governing NETs formation (33).

The formation of NETs is tightly regulated by a variety of microenvironmental factors. Existing reports indicate that multiple cytokines (e.g., GCSF, CXCL1, IL-8), hypoxia, and other stimuli can induce NETs formation (34–36). As a key activator of neutrophils, IL-8 (also referred to as CXCL8) shows a positive correlation between its expression level and NETs density in tumor tissues, and its transcription is regulated by a set of transcription factors including NF-κB (37), NFATc1 (38), NFATc2 (39), ATF3 (40), and WT1 (41). In addition, IL-17 promotes neutrophil recruitment and triggers NETosis through epithelial cell-derived signaling pathways (42), while glutamic acid-leucine-arginine (ELR)-positive chemokines promote NETs formation via the CXCR1/CXCR2 pathway (35).Beyond IL-8, GCSF is also regarded as a key inducer of NETs (6).

Cancer-associated fibroblasts (CAFs) are well-documented key inducers of NETosis (43), concurrently, NETs can promote the recruitment and activation of CAFs, forming a positive feedback loop that initiates micrometastasis of pancreatic ductal adenocarcinoma (44). In colon cancer, extracellular vesicles (EVs) derived from KRAS-mutant cancer cells can enhance NETs formation (45). Platelets regulate NETosis by forming platelet-neutrophil complexes (PNCs), and platelet dysfunction can lead to excessive NETs release and subsequent thrombosis (46). Notably, an alkaline microenvironment can promote NETs formation by enhancing calcium influx, mitochondrial ROS (mROS) production, PAD4 activity, and histone 4 cleavage (32).

Beyond their canonical antibacterial function, NETs play a pivotal role in regulating immune responses (3, 47). Specifically, they can promote the formation of an immunosuppressive microenvironment by mediating persistent inflammatory responses, thereby creating opportunities for tumor cell immune escape and ultimately facilitating tumor progression and metastasis (6).

## 4 Detection methods for NETs

### 4.1 ELISA

Enzyme-linked immunosorbent assay (ELISA) is widely employed for the quantitative analysis of NETs, particularly for the detection of specific markers such as citrullinated histone H3 (CitH3) and myeloperoxidase-DNA (MPO-DNA) complexes. Charlotte Thålin et al. established a highly specific, stable, and reproducible method for quantifying plasma CitH3, although its sensitivity partially depends on PAD4 activity (48). Subsequent refinements to the CitH3-DNA ELISA have further improved its reliability. While the MPO-DNA complex is commonly used for NETs quantification, its specificity in plasma samples has been questioned; therefore, the incorporation of isotype controls and a panel of NETs markers is recommended for robust assessment (49). A novel, highly sensitive ELISA, combined with immunofluorescence assay on smears, now enables the visualization of intact NETs structures from as little as 1 μL of serum or plasma, providing a simple, low-cost, and quantifiable alternative (50). Overall, ELISA remains a convenient and specific method, although challenges in standardization persist (51).

### 4.2 Immunofluorescence

Immunofluorescence microscopy is the classic approach for visualizing NETs through the staining of key components, including DNA, histones, and neutrophil elastase. This technique allows for the observation of structural changes and the discrimination between different NETosis stages and cell death modalities (21, 52). However, it is time-consuming and subject to operator-dependent interpretation.

### 4.3 Flow cytometry

Flow cytometry provides an objective and quantitative platform for NETs assessment. Gavillet et al. developed an antibody-based flow cytometric method targeting core NETs components—DNA, modified histones, and granule proteins, that is applicable to both human and mouse samples (53). Masuda et al. utilized SYTOX Green dye in combination with flow cytometry to achieve quantification equivalent to fluorescence imaging, yet with greater simplicity and objectivity (54). Zharkova et al. further validated a flow cytometry approach combining surface markers and nucleic acid staining in whole blood and purified polymorphonuclear cells, enabling high-throughput NETs detection in mixed cell populations (55). In summary, flow cytometry offers the advantages of rapidity, stability, and sort capability for NETs analysis.

### 4.4 Western blotting

Western blotting enables the visualization of NETs-related proteins, such as CitH3, with high specificity and sensitivity. However, certain antibodies are costly, require precise concentration optimization, and may exhibit non-specific binding in complex protein backgrounds (56, 57).

Translating NETs into clinical tools hinges on the stability and accuracy of detection methods. Commonly used techniques include fluorescence microscopy and ELISA for measuring MPO, cell-free DNA (CfDNA), CitH3, and MPO-DNA complexes; nevertheless, their clinical application remains constrained. Wargnies et al. developed an automated and standardized chemiluminescence immunoassay (ChLIA) for detecting H3.1-nucleosomes, an intrinsic NETs component, in plasma. This assay demonstrated high sensitivity, precision, and reproducibility, with validation across multiple NETs-associated diseases, indicating strong clinical potential (58). In active tuberculosis (ATB) research, bioinformatics and machine learning algorithms identified CD274, IRF1, and HPSE as NETs-derived diagnostic markers. These genes exhibit dual roles in neutrophil immunity and suggest therapeutic relevance, though further clinical validation is required (59).

In conclusion, the clinical reliability of NETs biomarkers depends not only on analytical performance but also on diagnostic validation in well-defined cohorts. Current evidence suggests that certain NETs components and gene combinations hold promise as robust clinical indicators; however, achieving routine application necessitates continued efforts in assay standardization and expanded clinical validation.

## 5 Neutrophil extracellular traps and tumor biology

### 5.1 NETs in modulation of the tumor microenvironment

A growing body of evidence supports that NETs are critical drivers of multiple hallmarks of tumor progression, including the reactivation of dormant tumor cells, adhesion of circulating tumor cells (CTCs), increased vascular permeability, and promotion of tumor metastasis and recurrence (6). For instance, a study by Song et al. demonstrated that high NETs expression in tumor tissues is significantly associated with adverse prognostic factors, such as larger tumor size, advanced tumor stage, poor differentiation grade, and vascular invasion. Additionally, elevated serum levels of myeloperoxidase-DNA (MPO-DNA) complexes — a well-recognized biomarker of NETs formation, not only contribute to tumor progression but also correlate with worse overall survival (OS) and progression-free survival (PFS) in patients (60). These findings collectively establish NETs as potential prognostic indicators and functional mediators of tumor progression.

Beyond their direct effects on tumor cells and vasculature, NETs play a pivotal role in reshaping the tumor immune microenvironment (61, 62). Transcriptomic analyses have revealed that neutrophil activation is significantly correlated with the expression of 17 NETs-related genes; these genes not only participate in the regulation of innate immune responses but also engage in functional crosstalk with adaptive immune cells (T cells, B cells) and innate lymphoid cells (natural killer cells) (63). Therefore, in-depth dissection of the mechanisms underlying interactions between NETs and immune cells will provide a critical theoretical foundation for unraveling the molecular basis of tumor progression and developing innovative immunotherapeutic strategies.

#### 5.1.1 T cells

T cells are classically recognized as the core effector cells of anti-tumor adaptive immunity; however, emerging evidence indicates that NETs undermine anti-tumor immunity and promote immune escape through multiple non-redundant mechanisms. First, NETs can directly induce T cell exhaustion, a state characterized by upregulated expression of inhibitory receptors (e.g., PD-1, CTLA-4), reduced secretion of effector cytokines(e.g., IFN-γ,TNF-α), metabolic reprogramming (e.g., enhanced glycolysis), and markedly impaired anti-tumor cytotoxicity (64). Second, and equally importantly, NETs can form a physical barrier around tumor cells, shielding them from recognition and killing by CD8^+^cytotoxic T cells (35). This dual mechanism direct functional impairment plus physical sequestration significantly compromises the anti-tumor efficacy of T cells, thereby facilitating tumor immune escape.

#### 5.1.2 Macrophages and dendritic cells

In regulating the function of antigen-presenting cells (APCs, including macrophages and DCs), NETs exhibit distinct time-dependent effects: short-term exposure (30minutes) induces the upregulation of co-stimulatory molecules (CD80 and CD86) on macrophages and DCs, thereby promoting their activation; in contrast, prolonged exposure triggers mitochondrial damage and subsequent apoptotic cell death (65). This time-dependent duality suggests that NETs may establish a dynamic and context-dependent immune regulatory network within the tumor microenvironment, with effects ranging from pro-inflammatory activation to immune suppression.

When co-stimulated with lipopolysaccharide (LPS), a canonical activator of toll-like receptor 4 (TLR4), NETs further modulate APC function by promoting inflammasome activation (as evidenced by increased IL-1β secretion) while inhibiting the production of anti-inflammatory (IL-10) and pro-immune (IL-12) cytokines (66). More notably, NETs can reprogram the differentiation trajectory of monocytes: they suppress the IL-4/GM-CSF-driven differentiation of monocytes into mo-DCs, key mediators of T cell activation, and instead skew differentiation toward anti-inflammatory (M2-like) macrophages (67). Given that M2-like macrophages are well-documented promoters of tumor angiogenesis, invasion, and immune suppression, this reprogramming capacity suggests a critical mechanism by which NETs contribute to tumor progression.

Mechanistically, studies have identified that NE, a key granular component of NETs, can inhibit the canonical signaling of protease-activated receptor 2 (PAR2) and regulate the miR-134a/miR-146a-CD24 axis to modulate the phagocytic function of macrophages (68). Notably, in a collagen-induced arthritis (CIA) model (a well-established model of chronic inflammation), DCs treated with NETs exhibit upregulated expression of maturation markers (e.g.,CD83, MHC II), increased IL-6 secretion, and enhanced Th1 cell responses (62). While this finding was observed in an autoimmune context, it highlights the pleiotropic effects of NETs on DC function, a property that may be hijacked by tumors to shape the immune microenvironment. Collectively, these observations provide new insights into the complex and context-dependent role of NETs in regulating APC function during tumor progression.

#### 5.1.3 NK cells

NK cells are a key subset of innate lymphoid cells and play a non-redundant role in tumor immune surveillance by recognizing and killing transformed cells in a MHC-independent manner (69). Recent studies have identified two distinct mechanisms by which NETs impair NK cell function: first, NETs can induce the upregulation of inhibitory molecules (LGAS9 and CEACAM1) on NK cells, directly suppressing their cytotoxic activity (63); second, CG, a serine protease enriched in NETs, can cleave NKp46, a critical activating receptor on NK cells, leading to impaired secretion of IFN-γ and reduced degranulation (a hallmark of NK cell-mediated killing) (70). Intriguingly, similar to their effect on CD8^+^T cells, NETs can also form a physical barrier around tumor cells to evade NK cell-mediated killing (35). These complementary mechanisms collectively enable tumor cells to escape innate immune surveillance mediated by NK cells.

#### 5.1.4 B cells

In anti-tumor immunity, B cells contribute to immune responses through multiple mechanisms, including the secretion of tumor-specific antibodies, the activation of T cells via antigen presentation, and direct cytotoxicity against tumor cells (71). Emerging evidence indicates that NETs can modulate B cell function through three key pathways: (1) NETs directly trigger antibody production in human memory B cells, wherein the LL-37-DNA complex (a component of NETs) activates the toll-like receptor 9 (TLR9) signaling pathway, a critical pathway for B cell activation and antibody class switching (72); (2) NETs promote B cell activation and the secretion of autoantibodies, which may contribute to chronic inflammation and tumor progression in certain contexts (61); (3) NETs facilitate the differentiation of B cells into plasma cells (antibody-secreting cells) via activation of the p38 MAPK signaling pathway (73). These findings suggest that NETs can reshape B cell-mediated immune responses, with potential dual roles in tumor progression (e.g., via pro-inflammatory autoantibodies) and anti-tumor immunity (e.g., via tumor-specific antibodies), a duality that requires further investigation in tumor-specific contexts.

**Figure 2:**
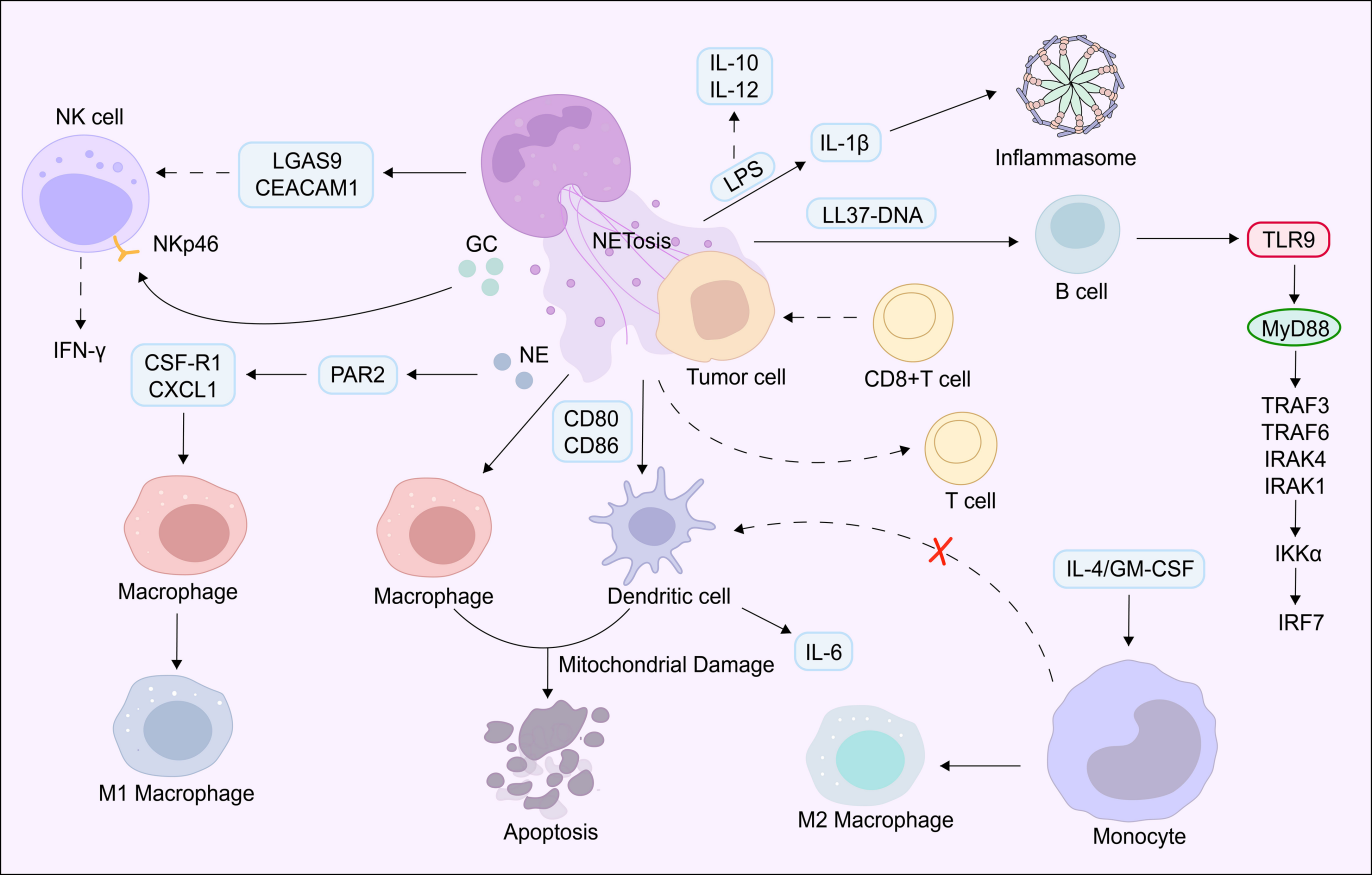
Summary of neutrophil extracellular traps in relation to immune cells. NETs promote tumor immune escape by inducing T cell exhaustion, impairing APC function, suppressing NK cell activity, and modulating B cell differentiation, thereby fostering a dynamic immunosuppressive microenvironment.

While existing evidence confirms an intimate and functional link between NETs and immune cells within the tumor microenvironment, critical gaps remain in our understanding of this relationship. For example, the context-dependent effects of NETs and the specific molecular determinants that govern these outcomes are not fully characterized. Future studies should focus on clarifying the tissue-and tumor-type-specific molecular pathways underlying NETs-immune cell interactions, with the goal of advancing the development of precision therapeutic strategies that target NETs to restore anti-tumor immunity (**Figure 2****).**

### 5.2 Metabolic regulation of NETs in gastrointestinal cancer TME

Emerging evidence indicates that metabolic reprogramming is a hallmark of cancer, supporting tumor cell proliferation, survival, and metastasis (74). The formation of NETs is highly dependent on metabolic pathways, with glycolysis playing a critical role and glutamine metabolism also contributing. For instance, PMA-induced NETosis proceeds in two phases: early chromatin decondensation occurs independently of exogenous glucose, whereas subsequent NET release is strictly dependent on both exogenous glucose and glycolysis (75). Conversely, NETs have been shown to upregulate lactate dehydrogenase A (LDHA), thereby driving glycolytic reprogramming and EMT in cancer cells. This highlights a reciprocal immune-metabolic interaction that presents a promising avenue for dual-targeting therapeutic strategies (76). In the context of a high-fat diet, PPARα deficiency exacerbates mitochondrial oxidative stress, activating the cGAS-STING-NF-κB pathway and promoting NET release. These NETs entrap T cells and NK cells, converting TME from “hot” to “cold” and consequently impairing antitumor immunity (77). Additionally, Chen et al. demonstrated that downregulation of ECI2 in colorectal cancer cells reshapes lipid metabolism, fostering NETs formation and accelerating tumor progression and metastasis. This finding deepens the understanding of the interplay between metabolic reprogramming and neutrophil biology in colorectal cancer (78). Recent studies further underscore a bidirectional regulatory relationship between NETs and metabolic reprogramming. NETs induced by surgical stress enhance fatty acid uptake and oxidative metabolism in cancer cells, thereby promoting metastatic growth. Concurrently, glycolytically reprogrammed neutrophil subsets, such as those with high CXCR4 expression, utilize the pentose phosphate pathway to generate NADPH, which fuels NADPH oxidase-dependent NETs formation. This establishes a metabolic–functional coupling in neutrophils (79).

In summary, tumor metabolic reprogramming and NETs formation mutually reinforce each other, collectively contributing to TME remodeling, EMT, and immune evasion. A deeper understanding of the mechanisms underlying this crosstalk may provide a theoretical foundation for developing combination therapies that concurrently target metabolism and NETs in gastrointestinal cancers.

### 5.3 Neutrophil extracellular traps in tumor onset and progression

In gastrointestinal tumors, the heterogeneity of NETs is evident throughout the entire process of tumor initiation and progression, manifesting as distinct molecular mechanisms driving NETs formation in different tumor types. First, not all neutrophils possess an equal capacity to induce NETosis. For instance, a PPIF^+^ neutrophil subset identified in colorectal cancer has been shown to drive pathological NETosis via the mitochondrial reactive oxygen species pathway (80). Second, the expression profiles of NETs-related genes exhibit marked diversity across tumor types, with key molecules such as SPP1 in liver cancer (81), HMGB1 in gastric cancer (82), SKAP1 in colorectal cancer (83), and TLR2 in pancreatic cancer (84) playing distinct roles in regulating tumor growth, immune suppression, and patient prognosis. Moreover, NETs tend to accumulate in specific microenvironmental niches. For example, at the tumor–stroma interface in hepatocellular carcinoma, SPP1^+^ M2-type macrophages co-localize with NETs-associated neutrophil signatures, forming a high-risk region that promotes tumor progression (85). This multidimensional heterogeneity underscores the need to shift future precision therapeutic strategies from broadly inhibiting NETs toward targeting specific NETs subtypes, key driver molecules, or their spatial niches. Achieving this goal will require an in-depth understanding of the mechanisms underlying NETs induction in the gastrointestinal tumor microenvironment and elucidation of how NETs regulate tumor progression, metastasis, and immune evasion through complex signaling networks.

NETs exhibit a complex dual role in tumor progression. On one hand, they can drive tumor metastasis by modulating the tumor immune microenvironment, promoting angiogenesis, and establishing pre-metastatic niches. On the other hand, under specific conditions, NETs exert antitumor effects, primarily through direct cytotoxicity against tumor cells and activation of immune responses (86). For instance, Chan et al. demonstrated in a pancreatic cancer mouse model that melatonin promotes the polarization of TANs toward an antitumor phenotype, facilitating ROS-dependent NETs formation and subsequently inducing tumor cell apoptosis (87). Furthermore, Shen et al. conducted a pan-cancer analysis of the prognostic value of NETs-related gene expression. Their findings revealed that the correlation between NETs score and patient survival is cancer type-dependent: higher NETs scores were associated with better survival outcomes in prostate, esophageal, breast, and colon cancers, whereas the opposite trend was observed in pancreatic cancer, lung squamous cell carcinoma, low-grade glioma, ovarian cancer, gastric cancer, and bladder cancer (88). Although NETs display tumor-suppressive effects in certain contexts, accumulating evidence suggests that their pro-tumorigenic roles are predominant. Therefore, an in-depth dissection of the molecular mechanisms underlying NETs function in tumor progression may provide novel therapeutic targets and a theoretical foundation for cancer treatment.

The association between NETs and tumors was first documented in Ewing’s sarcoma, where the presence of NETs correlates significantly with poor patient prognosis (89). While neutrophil infiltration and NETs formation in solid tumors are generally linked to adverse prognostic outcomes, the specific mechanisms underlying their pro-tumorigenic actions remain incompletely elucidated and require further investigation.

Emerging evidence suggests that these pro-tumorigenic effects may be mediated, at least in part, through metabolic reprogramming of cancer cells. Notably, NETs may drive tumor progression by regulating the energy metabolism of tumor cells. In suspended cancer cells, NETs enhance mitochondrial biogenesis and activate the TLR4-p38-PGC-1α signaling axis, which increases energy production in cancer cells and ultimately accelerates tumor growth (90). Collectively, a growing body of evidence indicates that NETs play a critical role in the onset, progression, and immune evasion of multiple tumor types—highlighting their potential as a conserved therapeutic target across cancer subtypes (**Table 1**).

**Table 1:** Regulatory mechanisms of neutrophil extracellular traps in tumor onset and progression.

Cancer type	Related Factors	Mechanism of Action	Effects	References
Hepatocellular carcinoma	S100A9	TLR4-ROS signaling pathway	Promotes NETs formation and HCC metastasis	(94)
	Acetyl-CoA	Activates CXCL1 epigenetically, recruits TANs	Promotes NETs formation and HCC metastasis	(96)
	TMCO6	Binds to NET-DNA, promotes apoptosis and TGFβ1 secretion	Facilitates NETs formation, immunosuppression, and HCC metastasis	(60)
	PPARα	Inhibits mitochondrial oxidative stress and CGAS-STING-NF-κB pathway	Suppresses NETs generation, enhances anti-tumor immune response	(77)
	SPP1	CD44/STAT3/CXCL1 axis	Promotes NETs formation and HCC metastasis	(81)
	HRG	Inhibits IL8-MAPK and NF-κB pathways, reduces ROS production	Suppresses NETs formation and HCC metastasis	(99)
	Nilotinib	Col1-DDR1-NFκB-CXCL8 axis	Inhibits NETs formation, improves immunosuppressive microenvironment	(97)
	PRSS35	Degrades CXCL2	Suppresses NETs generation and HCC progression	(101)
	Ozone	Activates AMPK, upregulates SR-A-mediated phagocytosis of DAMPs	Inhibits NETs formation and malignant ascites	(104)
Gastric Cancer	HMGB1	TLR4/p38 MAPK signaling pathway	Promotes NETs formation, enhances angiogenesis, and facilitates GC progression	(82)
	CCDC25	Promotes neovascularization and EMT	Facilitates GC metastasis	(111)
	TLR2	Activates COX-2	Facilitates GC metastasis	(112)
	IL8-CXCR1/2	Affects BRF1 p21/p27	Promotes GC progression	(113)
	SMYD2	NETs enhance NAT10-mediated ac4C modification of SMYD2, increasing its stability	Facilitates GC metastasis	(113)
	ANGPT2	Increased upon NETs stimulation	Promotes tumor proliferation	(115)
	TREM1	Facilitates NETs-mediated M2 macrophage polarization	Accelerates GC progression	(116)
	LIF	TGF-β-SMAD2/3-LIF axis	Promotes NETs formation and peritoneal metastasis	(118)
	LY2157299	TGF-β signaling pathway	Inhibits tumor metastasis	(122)
Colorectal Cancer	CD16^+^ neutrophils	CD16/TAK1/NF-κB axis	Reduces NK cell signaling, promotes NETs formation	(126)
	SKAP1	SKAP1-NFATc1-CXCL8 axis	Promotes NETs formation	(83)
	SPARC	Downregulation leads to neutrophil NADPH oxidase overactivation	Promotes NETs formation and accelerates tumor progression	(128)
	ARNT	Gut microbiota-CXCR2 signaling	Facilitates NETs formation and CRC progression	(129)
	TIMP1	Ferroptosis-related gene	Accelerates CRC progression	(130)
	SGK1	IL-6/STAT3/SAA signaling pathway	Promotes formation of pre-metastatic niche in liver metastasis	(136)
	FGF19	FGFR4-JAK2-STAT signaling pathway	Enhances NETs formation in liver metastatic microenvironment, accelerating liver metastasis	(139)
	KRAS	IL-8 overexpression	Promotes NETs formation and CRC progression	(142)
	ECI2	Inhibits AGPS, reducing ether lipid synthesis and IL-8 expression	Suppresses NETs formation and CRC progression	(78)
	Fn	Promotes EMT, basement membrane protein degradation, and CRC cell trapping	Facilitates NETs formation and tumor metastasis	(23)
	CEACAM1	Enhances CRC cell adhesion, migration, and metastasis	Promotes NETs formation and tumor metastasis	(26)
Pancreatic cancer	Thrombomodulin	Degrades HMGB1	Inhibits NETs formation and liver metastasis	(150)
	CAFs	Activated by NETs	Promotes liver micrometastasis	(44)
	DDR 1	DDR1/PKCθ/SYK/NF-κB signaling pathway	Increases CXCL5, promoting NETs formation and cancer cell invasion/metastasis	(151)
	NFE2	TGF-β/SMAD3 activates NFE2, regulating PAD4 transcription	Enhances NETs generation and tumor liver metastasis	(152)
	Obesity	Recruits and activates neutrophils	Promotes pancreatic carcinogenesis	(153)
	GAS	Streptococcus collagen-like protein 1	Suppresses tumor growth and NETs release	(154)

#### 5.3.1 Liver cancer

Hepatocellular carcinoma (HCC) is the most prevalent malignant tumor of the liver, characterized by high incidence and dismal prognosis (91, 92). As a specialized functional phenotype of neutrophils, NETs contribute substantially to the onset, development, and metastasis of HCC through multiple interconnected mechanisms.

Chronic hepatitis B virus (HBV) infection is a well-established driver of HCC onset, progression, and metastasis (93). Studies have demonstrated that HBV promotes NET formation by activating the S100A9-TLR4-ROS signaling pathway, thereby accelerating HCC progression. Furthermore, circulating NET levels may serve as a potential non-invasive biomarker for predicting extrahepatic metastasis in HBV-related HCC, offering clinical utility for risk stratification of patients (94).

The regulatory effects of NETs on HCC progression involve multiple molecular pathways. For instance, the m^6^A methyltransferase METTL5 is highly expressed in HCC tissues and accelerates the malignant progression of HCC by promoting NET release, linking epitranscriptional regulation to NET-mediated tumorigenesis (95). Additionally, TANs shape an immunosuppressive TME through NETs formation (8, 60, 90). Specifically, acetyl-CoA accumulation induces epigenetic activation of the chemokine CXCL1, which recruits TANs to the TME, triggers NET formation, and subsequently promotes HCC metastasis (96).NETs also directly modulate anti-tumor immunity: NET-DNA binds to TMCO6 on the surface of CD8^+^ T cells. This interaction inhibits the anti-tumor cytotoxic function of CD8^+^ T cells and promotes secretion of the immunosuppressive cytokine TGF-β1, thereby establishing a pro-tumor positive feedback loop that perpetuates immune evasion (60).

Metabolic abnormalities in the TME further influence the role of NETs in HCC. Deletion of PPARα enhances mitochondrial oxidative stress, activates the cGAS-STING-NF-κB pathway, and promotes NETs release. In turn, these NETs sequester T cells and NK cells, transforming the TME into an immunosuppressive “cold tumor” that is refractory to immunotherapy (77). Targeted intervention in NETs formation holds therapeutic potential for HCC. For example, nilotinib (a tyrosine kinase inhibitor) reduces NETs production by inhibiting the Col1-DDR1-NF-κB-CXCL8 signaling axis, which restores the anti-tumor activity of T cells and reverses TME immunosuppression (97).

Liver cancer metastasis is the leading cause of death in patients with HCC, and the formation of the PMN is a pivotal early event in the metastatic cascade. Thus, targeting the PMN represents an effective strategy to inhibit tumor metastasis (98). Studies have shown that SPP1 derived from HCC cells activates the CD44-STAT3-CXCL1 signaling axis in lung epithelial cells, promoting neutrophil recruitment and the formation of a neutrophil-enriched PMN, ultimately creating a permissive microenvironment for HCC lung metastasis (81). Additionally, HCC cells downregulate the expression of HRG to modulate the recruitment and activation of neutrophils in the metastatic microenvironment, thereby promoting NETs formation and lung metastasis (99). From a metabolic perspective, the glycolytic activity of neutrophils in HCC tissues is significantly increased and positively correlated with NETs release, suggesting that glycolytic inhibitors may represent a potential approach to suppress NET-mediated metastasis and improve patient outcomes (100). Moreover, PRSS35 reduces neutrophil recruitment and NETs formation by degrading CXCL2, highlighting its potential as a therapeutic target or diagnostic marker for HCC metastasis (101).

Malignant ascites is a common and debilitating complication of advanced HCC (102, 103), Recent studies have demonstrated that ozone activates AMPK, upregulates SR-A to enhance phagocytosis of DAMPs, and reduces NETs levels, thereby decreasing peritoneal fluid production. This finding provides a novel mechanistic insight into the treatment of advanced HCC-associated malignant ascites (104). Collectively, these observations establish NETs as central regulators of HCC pathogenesis and offer an important theoretical basis for the development of NETs-targeted diagnostic and therapeutic strategies for HCC.

#### 5.3.2 Gastric cancer

Gastric cancer (GC) is a globally prevalent malignant tumor, ranking among the top malignancies in terms of both incidence and mortality (105). Despite advancements in diagnostic technologies and therapeutic strategies, the 5-year survival rate for patients with advanced GC remains below 30% (106, 107), underscoring the need for novel therapeutic targets to improve clinical outcomes. In recent years, studies have consistently found that NETs are significantly enriched in the GC microenvironment and peripheral blood of GC patients. These NETs exhibit important diagnostic value and serve as independent prognostic factors for GC, with high NETs levels correlating with shorter overall survival (108), Additionally, NETs are associated with tumor heterogeneity and differential drug sensitivity in GC patients, implying their potential role in guiding personalized treatment decisions (109).

NETs regulate the apoptosis and invasion of GC cells by modulating the expression of Bcl-2 (an anti-apoptotic protein), Bax (a pro-apoptotic protein), and the NF-κB signaling pathway (110). Notably, current evidence suggests that NETs do not directly promote the proliferation of GC cells, but rather exert pro-tumor effects through indirect mechanisms. In the hypoxic TME (a hallmark of advanced GC), HMGB1 induces NETs formation via the TLR4/p38 MAPK pathway. These NETs enhance the migration and invasion capabilities of GC cells and indirectly support tumor growth by promoting angiogenesis (82). Similarly, NETs can activate endothelial cells via the CCDC25 receptor, thereby promoting angiogenesis and EMT, a key process driving tumor metastasis (111). At the molecular level, multiple signaling axes mediate NETs-induced EMT and tumor metastasis, including the SERPINE1-PAI-1-TGF-β axis and the TLR2-COX-2 pathway (112), Additionally, the IL8-CXCR1/2 axis, BRF1, cyclins p21/p27 (113), and NAT10-mediated N^4^-acetyl cytidine modification of SMYD2 (114) have all been implicated in this process. Furthermore, NETs-induced upregulation of angiopoietin 2 (ANGPT2) and TREM1-mediated M2 macrophage polarization (a source of immunosuppressive cytokines) further accelerate GC progression (115, 116). Recent research has also identified NETs-related lncRNAs as novel regulators of GC progression—opening a new direction for prognostic evaluation and therapeutic targeting in GC (117).

The peritoneum is the most common site of metastasis in advanced GC, and its microenvironment actively promotes neutrophil recruitment and NETs formation. Leukemia inhibitory factor (LIF) is an immunomodulatory signaling molecule; activation of the TGF-β-SMAD2/3-LIF axis induces neutrophil infiltration and NETs release in the peritoneal cavity, thereby driving peritoneal metastasis. This pathway thus represents a potential therapeutic target for preventing GC peritoneal dissemination (118). Radical gastrectomy combined with D2 lymph node dissection is the standard surgical treatment for locally advanced GC (119), however, the postoperative recurrence rate remains as high as 20–60% (120, 121), limiting long-term survival. Notably, postoperative abdominal infectious complications (AIC) stimulate NETs release, which promotes GC recurrence and metastasis via the TGF-β signaling pathway. TGF-β inhibitors may mitigate this risk, suggesting a potential role for adjuvant anti-TGF-β therapy in GC patients with postoperative AIC (122). Additionally, a predictive model based on the NETs score has been developed to evaluate patient prognosis, TME immune infiltration, and treatment response. This model provides a novel strategy for individualized GC therapy, enabling clinicians to stratify patients and select optimal treatment regimens (123). In summary, NETs accelerate GC progression through mechanisms such as promoting angiogenesis and immunosuppression. Targeting NETs or their regulatory pathways may improve patient prognosis; however, the specific mechanisms of NETs action in GC and their clinical translation require further investigation.

#### 5.3.3 Colorectal cancer

Colorectal cancer (CRC) is a prevalent malignancy with a high global disease burden (124). Its pathogenesis is closely linked to abnormal NETs formation in the tumor microenvironment (TME), which promotes CRC progression from initiation to metastasis (125). A key mechanism involves CD16^+^neutrophils, which induce cholesterol metabolism disorders via the CD16/TAK1/NF-κB axis, impairing NK cell function and promoting NETs release, offering novel diagnostic and therapeutic targets (126).

Mechanistically, SKAP1 promotes neutrophil recruitment and NETs formation via NFATc1/CXCL8; blocking this pathway inhibits CRC growth and enhances NK cell immunotherapy (83). NETs also drive invasion and metastasis by inducing EMT in tumor cells (127). SPARC regulates NADPH oxidase; its downregulation exacerbates NETs formation via integrin α5β1, accelerating CRC progression (128). ARNT modulates the gut microbiota-CXCR2 axis to influence NETs formation, linking microbiota to CRC progression (129). A recent multi-omics study further identified TIMP1 as a key regulatory gene for NETs formation in CRC; TIMP1 may contribute to CRC onset and development via the ferroptosis pathway, expanding our understanding of the crosstalk between NETs and cell death mechanisms (130).

The liver is the primary organ for CRC metastasis, and CRC liver metastasis (CRLM) is the leading cause of CRC-related death (131). Studies have shown that the immune microenvironment of CRLM is characterized by marked neutrophil infiltration (132), with NETs formation playing a pivotal role in driving the metastatic process. Tang et al. constructed a NETs-based gene prediction model for CRLM and identified CYP4F3 as a risk factor for liver metastasis and a potential intervention target (133). Additionally, highly metastatic CRC cells can induce NETosis in target organs prior to colonization, creating a permissive pre-metastatic niche (PMN) that supports subsequent tumor cell survival and outgrowth (16, 134, 135). At the molecular level, SGK1 promotes the formation of the CRLM PMN via the IL-6/STAT3/SAA signaling pathway. NETs and polymorphonuclear myeloid-derived suppressor cells (PMN-MDSCs) participate in this process in an SGK1-dependent manner, suggesting that targeting SGK1 may alleviate CRLM under ischemia-reperfusion (IR) conditions (136).

CAFs, key components of the CRC TME, promote fibrosis, immunosuppression, and tumor spread by inducing NETs formation (137, 138). Specifically, the FGF19-FGFR4-JAK2-STAT3 signaling axis drives the polarization of hepatic stellate cells into inflammatory CAFs (iCAFs); these iCAFs then promote neutrophil recruitment and NETs formation via C5a and IL-1β, ultimately accelerating CRLM (139). NE released by NETs activates the ERK signaling pathway in CRC cells, enhancing cell migration; inhibiting NE significantly reduces CRLM, indicating that NE may serve as a therapeutic target for preventing CRC metastasis (140). KRAS mutations present in approximately 40% of CRC cases (141), can be transmitted via tumor-derived exosomes, inducing overexpression of IL-8 in recipient cells and subsequent neutrophil enrichment, which in turn promotes NETs formation and CRC progression (142).

Metabolic reprogramming and the gut microbiota further modulate NETs-mediated CRC progression. ECI2 inhibits CRC progression by suppressing AGPS-dependent ether lipid synthesis, which reduces IL-8 expression and NETs formation (78). Additionally, Fn, a gut bacterium closely associated with CRC, enhances CRC invasiveness via NETs, with mechanisms involving EMT induction, basement membrane degradation (mediated by MMP2/9), and direct tumor cell capture by NETs (23). CEACAM1 has been identified as a NETs-associated molecule; blocking CEACAM1 or knocking out the CEACAM1 gene inhibits CRC metastasis, highlighting its potential as a therapeutic target (26). Immune checkpoint inhibitors (ICIs) have been approved for the treatment of microsatellite instability-high (MSI-H) metastatic CRC (mCRC); however, the objective response rate of single-agent ICI therapy remains low, likely due to TME immunosuppression. Studies have shown that targeting NETs reduces CD8^+^ T cell exhaustion and enhances ICI responses in CRC (143), suggesting that combining NETs inhibition with immunotherapy may represent a promising future research direction to improve treatment efficacy.

Gut microbiota dysbiosis, characterized by reduced microbial diversity and an overabundance of pro-carcinogenic bacteria, is a hallmark of CRC (144). Accumulating evidence highlights a bidirectional regulatory relationship between the gut microbiota and NETs formation. Tan et al. demonstrated that Parasutterella excrementihominis, via its metabolites succinate (Suc) and 6-hydroxyhexanoic acid (6-HHA), activates the succinate receptor 1/G protein-coupled receptor 84 (SUCNR1/GPR84) signaling axis, inducing Gasdermin D (GSDMD)-dependent NETosis, thereby promoting the progression of ulcerative colitis (UC) and colitis-associated colorectal cancer (CAC). Targeting this bacterium or its metabolic axis may represent a potential therapeutic strategy for inflammation-driven CRC (145). Pan et al. further elucidated the mechanistic role of Escherichia coli in colorectal cancer liver metastasis (CRCLM). E. coli activates RIPK2 in neutrophils, facilitating HNRNPK recruitment and subsequent upregulation of ATF3/Relb transcription. This cascade enhances NCF4 expression, triggering p-MLKL-mediated NET release and ultimately promoting liver metastasis (146). Moreover, CRC patients frequently exhibit compromised intestinal barrier integrity. Bacterial translocation has been shown to modulate the PMN, a process accompanied by inflammatory cytokine release and NETs formation (147). This establishes a vicious cycle: bacterial translocation → inflammation → NETs formation → barrier disruption → exacerbated bacterial translocation, that perpetuates CRC progression. Collectively, these findings suggest that both NETs and gut microbiota composition may serve as potential clinical indicators for predicting CRC progression and metastasis. Future research should focus on elucidating the mechanistic roles of specific microbial species in distinct CRC subtypes and facilitate the translation of these insights into clinical interventions.

#### 5.3.4 Pancreatic cancer

Pancreatic cancer is one of the most aggressive solid tumors, with a 5-year survival rate of less than 10% (124). In recent years, accumulating studies have established that NETs play a critical role in the onset and development of pancreatic cancer from early tumorigenesis to advanced metastasis, making them a potential therapeutic target. Miller-Ocuin et al. confirmed that neutrophil-derived DNA (a core component of NETs) activates pancreatic stellate cells that produce excessive ECM in the pancreatic TME. This activation promotes the formation of a dense fibrous stroma (a hallmark of pancreatic cancer) and enhances tumor proliferation. Notably, in PAD4 gene-knockout mice, pancreatic tumor growth was slowed and invasiveness was reduced, suggesting that DNase therapy (which degrades NET-DNA) may reverse TME fibrosis and suppress tumor progression (148).

Further studies have revealed that NETs promote pancreatic cancer cell secretion of IL-8 via activation of the STING pathway. IL-8, in turn, recruits more neutrophils to the TME, forming a positive feedback loop that amplifies NETs formation and neutrophil infiltration, perpetuating the pro-tumor TME (149). In terms of metastasis, NETs drive pancreatic cancer metastasis by inducing EMT in tumor cells, enhancing their ability to invade surrounding tissues and colonize distant organs. Conversely, thrombomodulin (a glycoprotein with anti-coagulant and anti-inflammatory properties) inhibits NETs formation by degrading HMGB1, thereby reducing pancreatic cancer liver metastasis (150). Additionally, NETs activate CAFs in the liver microenvironment, promoting the formation of hepatic micro metastases (44). Deng et al. further clarified that the collagen/DDR1 signaling axis induces CXCL5 secretion in pancreatic cancer cells via the PKCθ/SYK/NF-κB pathway. CXCL5 then recruits TANs to the TME and induces their NETs formation, ultimately accelerating pancreatic cancer metastasis (151). Regarding the metastatic microenvironment, Xu et al. combined single-cell RNA sequencing and spatial transcriptomics to dissect the cellular and molecular landscape of pancreatic cancer liver metastasis. They found that the TGF-β/SMAD3/NFE2 pathway promotes NETs production by upregulating PAD4 expression in neutrophils, which directly drives liver metastasis (152). Wang et al. confirmed that obesity promotes NETs formation by recruiting neutrophils to the pancreatic TME, accelerating the onset and development of pancreatic cancer; this pro-tumor process can be reversed by metformin (a widely used anti-diabetic drug) and DNase I, providing a potential repurposing strategy for metformin in pancreatic cancer (153). Intriguingly, GAS inhibits NETs release via its collagen-like protein Scl1, offering an unexpected perspective for developing microbe-based therapeutic approaches to target NETs in pancreatic cancer (154). Collectively, these studies systematically delineate the key role of NETs in the onset, development, and metastasis of pancreatic cancer, and provide a solid theoretical basis for the development of NETs-targeted therapeutic strategies.

### 5.4 Neutrophil extracellular traps and tumor metastasis

Accumulating evidence has established that NETs play a pivotal role in tumor metastasis, a process that remains a major cause of cancer-related mortality. Multiple independent studies have demonstrated that elevated NETs levels correlate significantly with the occurrence of liver and lung metastasis in various cancer type (16, 155–157). The pro-metastatic effects of NETs are primarily mediated through three interconnected mechanisms: (1) forming complexes with tumor cells to facilitate ECM degradation, thereby promoting local invasion; (2) sustaining the survival of CTCs by protecting them from anoikis and immune surveillance; and (3) remodeling the distant microenvironment to establish the PMN (7–9). Collectively, these findings support the notion that targeting NETs may represent a novel and promising strategy for inhibiting tumor metastasis.

Two well-characterized mechanisms underlie NETs-induced stimulation of tumor cell migration. First, NET-DNA binds to the transmembrane receptor CCDC25 on tumor cells, which activates the ILK-β-parvin signaling pathway and subsequently enhances cell motility. Second, NETs can induce EMT in tumor cells, a phenotypic shift that endows cancer cells with increased invasive and migratory properties, a hallmark of metastatic competence (134). Liu et al. further validated the specificity of the NET-DNA/CCDC25 interaction, demonstrating that CCDC25 binds to NET-DNA with high affinity and selectivity. This binding event activates the ILK-Parvb-RAC1-CDC42 signaling cascade, which directly drives cytoskeletal reorganization and tumor cell migration. Notably, the researchers developed shCCDC25 delivery system using the oncolytic bacterium VNP. This system not only significantly inhibited lung metastasis in preclinical models but also modulated the infiltration of immune cells in the TME, highlighting its potential as a dual-acting anti-metastatic and immunomodulatory strategy (158). TME-derived factors, particularly hypoxia (a common feature of advanced tumors), can further promote NETs formation and accelerate the metastatic process. Hypoxia induces NETosis via PGC-1α-mediated enhancement of mitochondrial respiration in neutrophils, which increases ROS production—a key driver of NETs formation (90, 159). In preclinical gastric cancer models, NETs have been experimentally verified to induce EMT in tumor cells and promote liver metastasis, directly linking TME hypoxia, NETs formation, and organ-specific metastasis (111). In addition to distant organ metastasis, NETs also contribute to lymph node metastasis. Su et al. observed that neutrophil recruitment and NETs deposition occur at the early stages of lymph node metastasis in cancer models. This process is driven by the secretion of the chemokines CXCL8 and CXCL2, which are released by tumor-derived exosomes. These exosomes mediate intercellular communication between tumor cells and neutrophils, triggering NETs formation in the lymph node microenvironment and creating a permissive niche for metastatic colonization (160). Peritoneal metastasis (PM) is a common and devastating form of cancer dissemination, particularly in gastrointestinal malignancies. CRS is the primary therapeutic intervention for resectable PM (161, 162). However, CRS is often associated with adverse clinical outcomes, including postoperative tumor recurrence, distant metastasis, and peritoneal adhesion formation, complications that significantly reduce patient survival (163, 164). To address NETs-mediated complications following PM surgery, Wang et al. developed a novel polysulfoxide-based material, PMeSEA. This material exerts a dual therapeutic effect: it specifically scavenges hypochlorite (ClO^-^, a critical mediator of NETs formation) to inhibit NETosis, and exhibits significant efficacy in preventing postoperative peritoneal adhesions and suppressing metastatic progression. When combined with chemotherapeutic agents, PMeSEA enhances drug delivery to the peritoneal cavity and exerts a synergistic anti-tumor effect, further improving therapeutic outcomes (165). Taken together, these studies not only clarify the multifaceted roles of NETs in tumor metastasis but also provide valuable insights into the development of NETs-targeted therapeutic targets and strategies for the clinical intervention of metastatic cancer.

## 6 Potential applications of neutrophil extracellular traps in tumor treatment

### 6.1 Inhibition of NETs formation

PAD4 is a neutrophil-specific enzyme that catalyzes the citrullination of histones in a calcium ion (Ca²^+^)-dependent manner. This enzymatic reaction induces chromatin decondensation, a key step in promoting NETs formation (166–169). Emerging studies have demonstrated that PAD4 inhibitors (e.g., GSK484, YW4-03, JBI-589, and BMS-P5) can effectively reduce NETs production, thereby identifying PAD4 as a novel intervention target for tumor therapy (170–175).

In CRC, the PAD4 inhibitor GSK484 significantly enhances tumor radiosensitivity by promoting radiation-induced DNA damage and cancer cell apoptosis. Both *in vitro* and *in vivo* experiments confirmed that GSK484 not only inhibits NETs formation but also delays tumor growth (170). Similarly, in a triple-negative breast cancer (TNBC) model, combinatorial treatment with GSK484 and radiotherapy exerted a synergistic antitumor effect: this combination inhibited the proliferation, migration, and invasion of tumor cells while promoting apoptosis, suggesting that PAD4 inhibition may represent a viable strategy to improve the efficacy of radiotherapy for TNBC (173). Additionally, Shi and colleagues found that either PAD4 gene knockout or treatment with the PAD4 inhibitor YW4–03 can reduce NETosis, thereby suppressing the proliferation and metastasis of breast cancer cells (171). The novel PAD4 inhibitor JBI-589 exhibits multiple antitumor effects: it inhibits CXCR2-mediated neutrophil chemotaxis, reduces primary tumor growth and lung metastasis, and enhances the antitumor efficacy of ICIs (175). In multiple myeloma (MM), the PAD4 inhibitor BMS-P5 has also been shown to reduce NETs formation and delay disease progression (174).

NE exerts complex biological functions within the TME. On one hand, clinical studies have shown that NE inhibitors (e.g., sivelestat) can improve postoperative recovery in patients with esophageal cancer (176); on the other hand, NE has been reported to selectively kill tumor cells under specific conditions (177). This dual pro-tumor/anti-tumor role highlights the potential therapeutic value of NE in tumor treatment but also underscores the need for in-depth exploration of its precise mechanisms of action.

Recent studies have identified an unexpected role for GLP-1 analog liraglutide in NETs modulation: beyond its established function in blood glucose regulation, liraglutide reduces NETs formation by inhibiting the production of ROS, thereby enhancing the antitumor effect of immunotherapy. In lung cancer and liver cancer models, combinatorial treatment with liraglutide and PD-1 blockers significantly improved antitumor efficacy; this effect may be associated with the restoration of CD8^+^ T cell function, providing a novel insight for the development of combined ICI-based therapeutic strategies (178).

Furthermore, multiple studies have uncovered the regulatory roles of additional targets in NETs formation and their potential applications in tumor treatment: Downregulation of CD276 (a co-inhibitory molecule) can ameliorate the NETs-mediated immunosuppressive TME (179); Acetyl-coenzyme A (acetyl-CoA)-targeted therapy inhibits NETs formation by blocking the CXCL1-CXCR2 signaling pathway, thereby reducing HCC metastasis (96); Inhibition of HMGB1 reduces neutrophil activation and NETs release via the TLR4/p38 MAPK signaling pathway (82); Regulation of neutrophil recruitment and NETs formation by the SKAP1-NFATc1/CXCL8 signaling axis plays a critical role in CRC progression (83). These findings collectively provide an important theoretical basis for the development of NETs inhibition-based tumor treatment strategies.

### 6.2 Direct targeting of neutrophil extracellular traps

The primary structural component of NETs is extracellular DNA, which can be degraded by DNA-degrading enzymes (e.g., DNase-I) present in serum. DNase-I is an endonuclease that occurs naturally in plasma (180). In 1993, the U.S. Food and Drug Administration (FDA) approved recombinant human DNase-I (trade name: Pulmozyme) for the treatment of cystic fibrosis; its therapeutic mechanism involves the degradation of extracellular DNA accumulated in the lungs of affected patients (181). Recent studies have extended the potential applications of DNase-I to antitumor therapy. For example, a study by Tsung et al. demonstrated that adeno-associated virus (AAV)-mediated hepatic gene delivery of DNase-I effectively inhibits tumor progression in a murine model of CRC liver metastasis (182).

**Figure 3:**
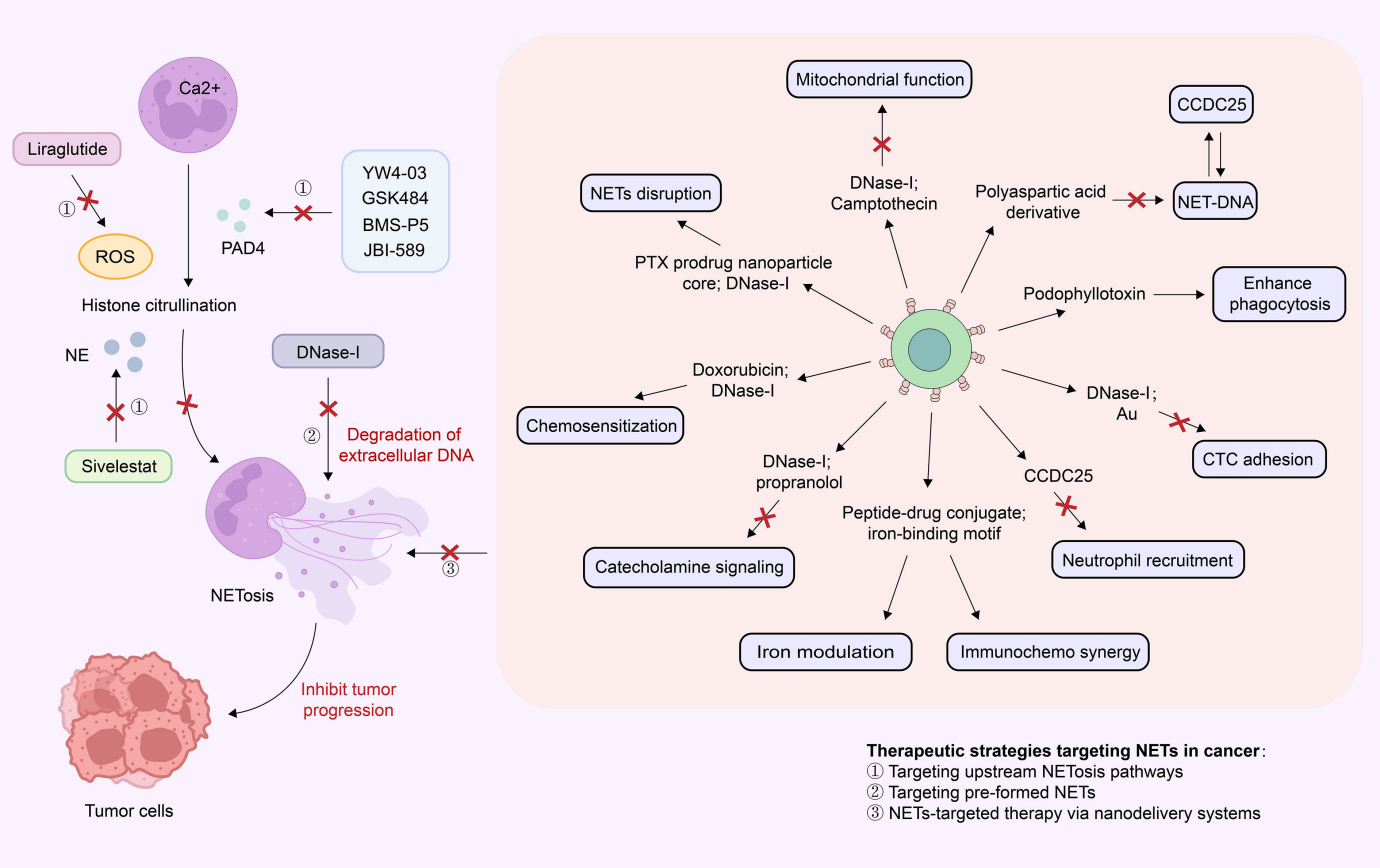
Targeting NETs for cancer therapy: mechanisms and strategies. This schematic illustrates therapeutic strategies targeting neutrophil extracellular traps (NETs) in cancer treatment. It clearly delineates the calcium-dependent, PAD4-mediated pathway of NETs formation, along with core therapeutic approaches for targeting NETs-including upstream inhibition (PAD4 inhibitors, NE modulation, GLP-1 analogs, etc.), downstream clearance (DNase-I-mediated extracellular DNA degradation), and nanoparticle delivery systems for enhanced therapy. By inhibiting NETs formation and/or degrading already released NETs, these strategies ultimately achieve suppression of tumor progression and enhance the efficacy of radiotherapy, chemotherapy, and immunotherapy.

In addition, nanoparticle-based drug delivery systems represent a novel strategy for NETs-targeted therapy, owing to their unique properties including modifiability, stimulus responsiveness, and active targeting capability. These systems can optimize drug delivery efficiency, enhance drug stability, and enable precise targeting of NETs, thereby improving overall antitumor efficacy (**Table 2****) (****Figure 3**).

**Table 2:** Nanoparticle delivery systems related to neutrophil extracellular traps.

Nanoparticle system	Loaded drug/Molecule	Mechanism of action	Model/Application	References
PTX	PTX prodrug nanoparticle core; DNase-I	Disrupts tumor-associated NETs	Suppresses pulmonary metastasis of tumors	(183)
DNase-I-loaded liposomes	CCDC25	Eliminates NETs and significantly inhibits neutrophil recruitment	Suppresses liver metastasis in colorectal cancer	(184)
Mycoplasma membrane (MM)-fused liposomes	Podophyllotoxin(POD)	Activates neutrophils to enhance phagocytosis of MM-modified liposomes, promoting targeted accumulation of nanodrugs	Significantly inhibits tumor growth and lung metastasis in a 4T1 breast cancer model	(185)
Polyaspartic acid-based cationic material	Polyaspartic acid derivative(cANP)	Interferes with NET-DNA and CCDC25 interaction	Suppresses cancer metastasis in murine and human metastatic models	(186)
DMMnSiO3-PEG/DOX/DNase-I	Doxorubicin; DNase-I	Synergistically degrades NETs and enhances chemotherapeutic efficacy	Inhibits breast cancer lung metastasis	(187)
DNAzyme I@Au	DNase-I; Au	Degrades ROS-induced NETs; prevents adhesion of circulating tumor cells to tumor sites or vasculature	Nebulized inhalation; enhances radiosensitivity; suppresses tumor growth and metastasis	(188)
Transformable iron nanocheator (TIN)	Peptide-drug conjugate; iron-binding motif	Modulates iron metabolism; inhibits NETs formation; synergizes with PAD4 inhibitors or anti-PD-L1 to enhance antitumor immunity	Reverses NETs-mediated immune evasion	(189)
5HT-NP@D + p-TC-RLA	DNase-I; Camptothecin (CPT)	1. Degrades NET-DNA scaffold and inhibits mitochondrial biogenesis; 2. Disrupts mitochondrial function, alleviates hypoxia, and reduces neutrophil infiltration	Suppresses spontaneous and disseminated metastatic tumor models in the lungs	(190)
Fibrin-alginate hydrogel	DNase-I; propranolol	Degrades NETs; Blocks catecholamine signaling	Prevents tumor recurrence and metastasis after incomplete SR	(191)

Studies have demonstrated that liposomal nanocarriers can significantly enhance the pharmacokinetic properties of DNase-I. While no significant differences in DNase-I activity were observed in *in vitro* experiments, optimally engineered liposomal carriers extended the half-life of DNase-I by up to 3-fold following intravenous administration in animal models (192). Additionally, research has identified that nucleosomes (NS) released during NETs formation by neutrophils can be specifically recognized by the monoclonal antibody 2C5. This finding suggests that 2C5 not only enables the detection and visualization of NETs in biological samples or tissues but also holds substantial application potential for the development of NETs-targeted therapeutic strategies (193).

NETs has emerged as a promising strategy in cancer immunotherapy. Accumulating evidence indicates that combining NETs inhibitors with immune checkpoint inhibitors, chemotherapy, or radiotherapy can enhance antitumor efficacy. Wu et al. reported that in non-immunogenic colorectal cancer, upregulation of FGF19 promotes neutrophil recruitment and NETs release, leading to suppression of CD8^+^ T cell function and facilitation of tumor progression. Notably, combination therapy with the FGFR4 inhibitor BLU-9931 and anti-PD-1 antibody sensitized microsatellite-stable (MSS) colorectal cancer, which is typically resistant to immunotherapy, to immune checkpoint blockade (194). In PDAC, NETs-associated arginase 1 (ARG1) suppresses T cell activity. Treatment with a specific ARG1 monoclonal antibody combined with immune checkpoint inhibitors restored CD8^+^ T cell function *in vitro* and enhanced responsiveness to immune checkpoint therapy in a humanized mouse model (195). Chemotherapeutic agents often induce neutrophil recruitment and NETs formation, which may contribute to therapeutic resistance. For instance, in a PDAC model, the addition of DNase to the gemcitabine plus nab-paclitaxel (GNP) regimen significantly enhanced tumor suppression and prolonged survival (196). However, Li et al. revealed a potential antitumor role of chemotherapy-induced NETs: the combination of the glutaminase inhibitor CB-839 and 5-FU elevated ROS levels in neutrophils, triggering NETs release and enabling cathepsin G (CTSG) to enter tumor cells and induce apoptosis, thereby suppressing tumor growth in a colorectal cancer mouse model (197). This study further underscores the dual role of NETs in cancer, highlighting the need for context-dependent strategies in NETs-targeted therapies.

### 6.3 Neutrophil extracellular traps and tumor drug resistance

**Figure 4:**
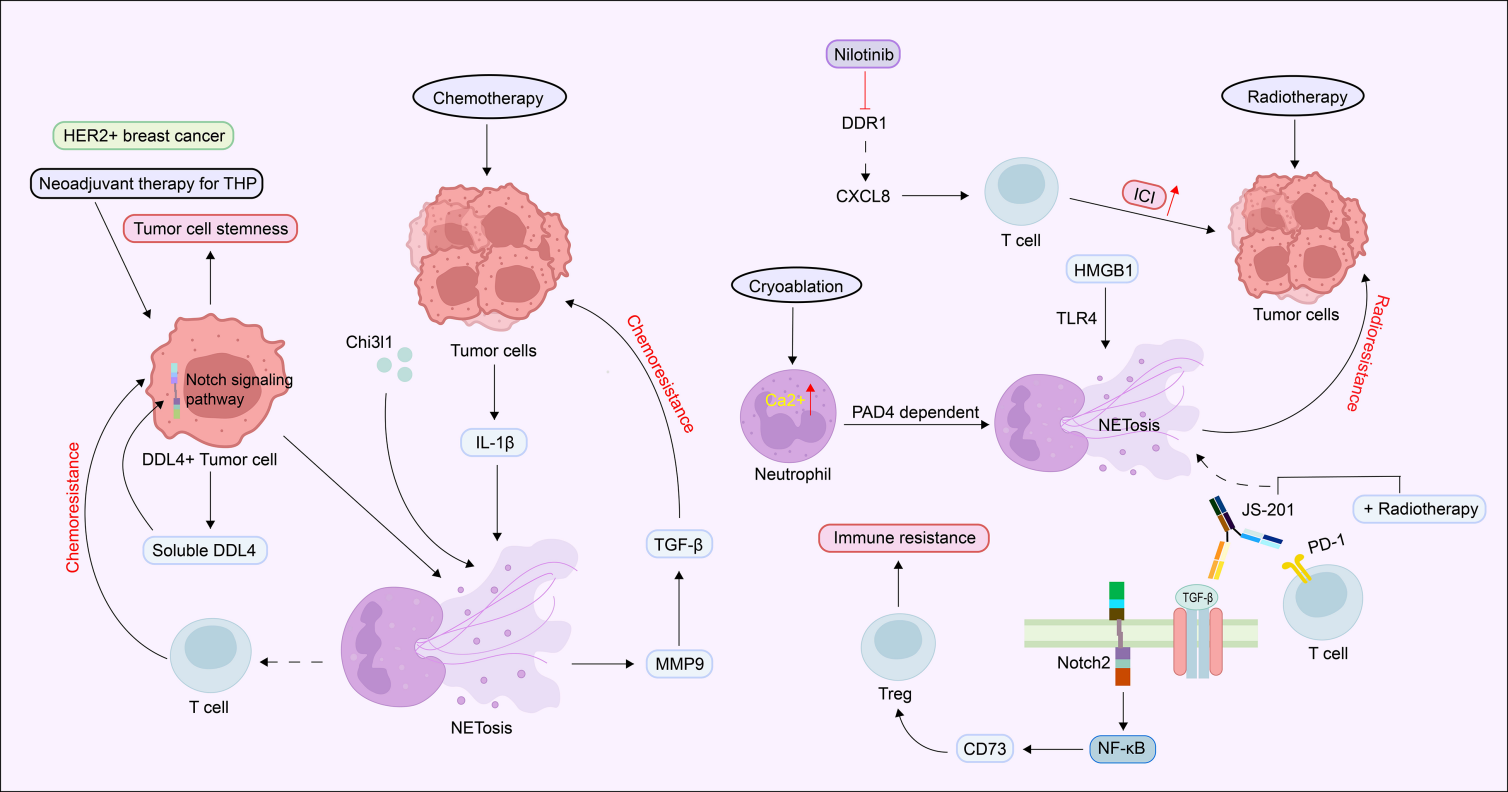
NETs-associated mechanisms of tumor therapy resistance. Chemotherapy and radiotherapy promote NETs formation through IL-1β/TGF-β/MMP9, DLL4-Notch, and HMGB1-TLR4 pathways, contributing to resistance to chemo/radiotherapy and immunotherapy in breast cancer and other malignancies. Targeting NETs-associated pathways (e.g., DLL4-CAR-T, PD-1/TGF-β bispecific antibodies) may reverse treatment resistance.

NETs play a pivotal role in driving resistance to various anticancer therapies. In breast cancer, chemotherapy-induced interleukin-1β (IL-1β) promotes NETs formation through activation of the TGF-β and MMP9 signaling pathways, thereby contributing to chemoresistance. Notably, NETs-mediated proteolytic activation of TGF-β represents a key mechanism by which cancer cells acquire resistance to chemotherapy (198). These findings suggest that NETs can amplify pro-tumor signaling cascades and confer a drug-resistant phenotype. In HER2-positive breast cancer, tumor cells with high expression of DLL4 exhibit stem-like properties and resistance to neoadjuvant therapy comprising trastuzumab, pertuzumab, and paclitaxel. Mechanistically, tumor-derived soluble DLL4 activates Notch signaling in neutrophils, inducing NETs formation and reducing lymphocyte infiltration. Targeted elimination of DLL4^+^tumor cells using DLL4-directed CAR-T cells effectively reversed this resistance (199). Similarly, NETs have been shown to promote regulatory T cell infiltration via the Notch2/NF-κB/CD73 axis, thereby facilitating immune evasion in hepatocellular carcinoma (200). In the context of radiotherapy, NETs accumulation is also associated with resistance. In a mouse model of bladder cancer, significant NETs deposition was observed following irradiation, and HMGB1 promoted NETs formation through the TLR4 pathway, contributing to radioresistance. Combination therapy with the PD-1/TGF-β bispecific antibody JS-201 attenuated NETs release and enhanced the antitumor efficacy of radiotherapy (201, 202). Furthermore, multiple studies have established a link between NETs and resistance to immune checkpoint inhibitors. Key findings include the following (1): the DDR1 inhibitor nilotinib improved immunotherapy responses in hepatocellular carcinoma by modulating the Col1-DDR1-CXCL8 axis (97); (2) the CXCL2-CXCR2-Ca²^+^-PAD4 signaling axis influenced the efficacy of PD-1 antibody therapy following cryoablation (203); and (3) complement C5a-induced NETs from myeloid-derived suppressor cells (MDSCs) impaired the efficacy of PD-1/PD-L1 blockade (204) (**Figure 4**).

Collectively, NETs contribute to therapeutic resistance through multiple mechanisms, including direct activation of intracellular signaling pathways in tumor cells (e.g., TGF-β), remodeling of the immunosuppressive tumor microenvironment, and formation of physical barriers. Importantly, chemotherapy and radiotherapy themselves can induce NETs formation, which in turn exacerbates resistance to subsequent treatments, including immune checkpoint inhibitors. A deeper understanding of the mechanisms underlying NETs-mediated therapy resistance will inform the development of rational combination strategies to improve therapeutic outcomes.

### 6.4 Clinical translation of NETs-targeted therapies

Although NETs play a significant role in the pathogenesis of various diseases, the clinical translation of therapies targeting NETs remains challenging. Currently, specific drugs targeting NETs are still in preclinical stages, with no publicly available human clinical data. This limitation stems largely from the complexity of NETs formation mechanisms and their substantial heterogeneity in tumors, which complicates the precise prediction and monitoring of their dual pro- and anti-tumor effects.

#### 6.4.1 Intrinsic complexity

NETs are highly dynamic and heterogeneous structures composed of a DNA scaffold and various proteins. Their morphology and protein composition vary depending on disease type and stage (4), rendering strategies targeting a single component largely ineffective. Moreover, neutrophils can undergo NETosis through multiple signaling pathways (22), the same stimulus may trigger NETs release via distinct mechanisms, making it difficult to completely inhibit NETs formation by blocking a single pathway. Additionally, the short survival time of neutrophils *in vitro* (typically only a few hours) limits the assessment of long-term effects of NETs inhibitors (205, 206).

#### 6.4.2 Limitations of preclinical models

NETs inhibitors that show significant efficacy in animal models often fail to produce comparable results in humans, highlighting the inadequacies of current models. First, neutrophils constitute the highest proportion of leukocytes in humans, whereas in mice, they account for only approximately 20% (2). Second, morphological differences exist between human and murine NETs: murine NETs are more compact, whereas human NETs exhibit a more expanded and reticular structure (207). Furthermore, most preclinical studies utilize acute disease models in healthy mice, which fail to replicate the complex, long-term, and multifactorial pathological environment observed in human patients.

#### 6.4.3 Challenges in drug targeting

Although DNase-I can degrade NETs, its low stability in serum and susceptibility to inactivation by heat and environmental stimuli pose challenges for maintaining effective concentrations and sustained activity at lesion sites (208). Additionally, many protein components of NETs are involved in normal physiological functions. Thus, achieving a balance between inhibiting pathological NETs formation and preserving their physiological defensive functions remains a critical challenge in therapeutic strategy design.

## 7 Discussion

Neutrophil extracellular traps have emerged as a pivotal focus in cancer research, owing to their multifaceted roles in tumorigenesis, progression, and therapeutic resistance. Recent studies have demonstrated that NETs contribute to malignant progression through multiple mechanisms: on one hand, they facilitate tumor metastasis by inducing EMT and angiogenesis in tumor cells; on the other hand, they promote tumor immune evasion by remodeling the tumor immune microenvironment, for instance, by suppressing T-cell function. Therapeutically, NETs confer resistance to radiotherapy, chemotherapy, and immunotherapy via the formation of physical barriers and modulation of molecular signaling pathways. Although NETs-targeting strategies have exhibited considerable therapeutic potential in preclinical models, their translation into clinical practice is hindered by several challenges and limitations.

First, NETs appear to exert a dual role in tumor biology: they may support early-stage tumor immune surveillance while promoting late-stage tumor immune evasion. However, the precise regulatory mechanisms underlying this duality remain elusive. Second, given the dual functions of NETs in infection defense and tumor progression, the development of strategies to selectively target NETs in the tumor microenvironment without compromising the host’s anti-infection immunity, requires further investigation. Additionally, current methods for NETs detection predominantly rely on indirect assays and the lack of specific, reliable biomarkers for NETs has impeded the progress of clinical research in this field.

Future studies should leverage advanced technologies, including single-cell sequencing and spatial transcriptomics to delineate the heterogeneity of NETs and their interaction networks within the tumor microenvironment. Furthermore, the development of highly specific molecular imaging probes and dynamic NETs monitoring techniques is crucial for evaluating the clinical relevance of NETs in cancer patients. Additionally, exploring precision NETs-targeted therapies, particularly their synergistic effects with existing cancer treatments—may uncover novel strategies for improving cancer intervention outcomes.

## 8 Conclusion

In summary, the role of NETs in tumor biology is highly complex, with context-dependent pro-tumor and anti-tumor effects. In-depth elucidation of the mechanisms underlying NETs formation, function, and regulation is expected to represent a new breakthrough in understanding tumor initiation, progression, and immune evasion. This line of research not only provides novel therapeutic targets and conceptual frameworks for advancing tumor immunotherapy but also holds promise for driving substantial improvements in the prognosis and clinical outcomes of patients with cancer.
